# Comparative analysis of virulence factors produced by *Pseudomonas aeruginosa* strains isolated from chronic wounds or bloodstream infections

**DOI:** 10.17912/micropub.biology.001413

**Published:** 2024-12-18

**Authors:** Felipe L. Teixeira, Heidi Pauer, Gabriel Luis C. Valente, Geraldo Renato de Paula

**Affiliations:** 1 Department of Molecular Biosciences, University of Kansas, Lawrence, Kansas, United States; 2 Departamento de Tecnologia Farmacêutica, Universidade Federal Fluminense, Niterói, Rio de Janeiro, Brazil; 3 Instituto Nacional de Ciência e Tecnologia de Inovação em Doenças de Populações Negligenciadas, Fundação Oswaldo Cruz, Rio de Janeiro, Rio de Janeiro, Brazil

## Abstract

*Pseudomonas aeruginosa* is an important pathogen associated with both chronic wounds and bloodstream infections. Virulence factors required for the establishment of acute and chronic infections differ substantially. Since bacteremia can be a severe outcome of wound colonization, we performed a comparative analysis of virulence between *P. aeruginosa* strains isolated from the bloodstream and chronic wounds. Our results show that, in general, *P. aeruginosa* strains isolated from bloodstream infections are more virulent than strains that colonize chronic wounds.

**Figure 1. Comparison of virulence phenotypes between P. aeruginosa strains isolated from bloodstream infections or chronic wounds.**
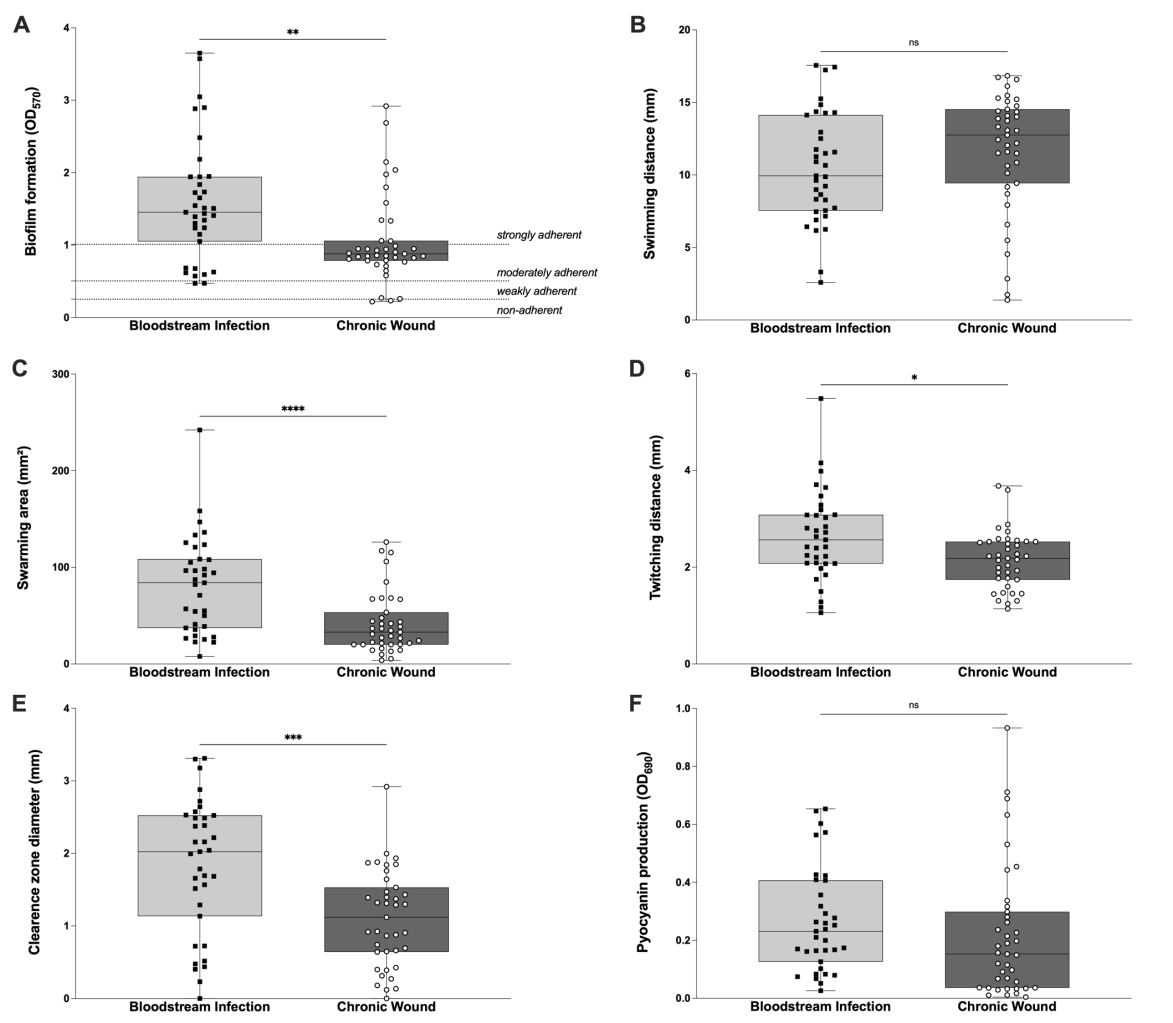
We analyzed the phenotypic expression of virulence factors of 74 *P. aeruginosa* strains previously isolated from bloodstream infections or chronic wounds. (A) Overnight biofilms were stained with 0.1% crystal violet and the optical density (OD) of each well was measured at 570 nm (OD _570_ ) to determine the biofilm formation capability of each strain. Based on the OD _570_ measurements, strains were also classified into four categories: non-adherent, weakly adherent, moderately adherent, and strongly adherent. Overall, strains isolated from bloodstream infections formed stronger biofilms than strains from chronic wounds. (B) Swimming motility was evaluated by measuring the diameter of colonies grown in LB plates with 0.3% agar for 24 hours. (C) Swarming motility was evaluated by measuring the area of colonies grown in LB plates with 0.6% agar for 24 hours. (D) Twitching motility was evaluated by stabbing *P. aeruginosa* strains into the agar of LB plates with 1% agar and, after 24 hours, staining the bottom of plates with 0.1% crystal violet. The diameter of the stained area represented the twitching distance of each strain. (E) Proteolytic activity was analyzed by measuring the clearance zone diameter around colonies grown for 24 hours in LB agar with 2% skimmed milk. (F) Pyocyanin production was measured by chloroform extraction from 48-hour cultures and quantification in a spectrophotometer at an OD of 690 nm (OD _690_ ). The box in each graph extends from the 25th to 75th percentiles and the line in the middle is plotted at the median. Each experiment was performed in triplicate and symbols represent the mean values of the replicates for each *P. aeruginosa* strain. Whiskers are drawn from the smallest to the largest value. Significance between groups was analyzed using the Mann-Whitney test. ns: p> 0.05; *: p ≤ 0.05; **: p ≤ 0.01; ***: p ≤ 0.001; ****: p ≤ 0.0001.

## Description

*Pseudomonas aeruginosa* is a common opportunistic pathogen in hospital settings that causes a wide range of acute and chronic infections, both of great concern to human health (Valentini et al., 2018; Qin et al., 2022) . Infection of the epithelial tissue by *P. aeruginosa* is often associated with injuries such as burns or chronic wounds (Ruffin and Brochiero, 2019) . The latter is of particular concern since these non-healing wounds are a serious socioeconomic burden to healthcare, with an estimated cost of over 12 billion dollars (Kolimi et al., 2022) . Bacterial colonization is a major contributor to wound chronicity (Ammons et al., 2015) and *P. aeruginosa* is one of the species more frequently isolated from such wounds (Phan et al., 2023) . Virulence factors produced by colonizing *P. aeruginosa* can result in epithelial damage and may contribute to impaired wound healing (Ruffin and Brochiero, 2019) .

A severe outcome of wound colonization that can put the patient's life at risk is the translocation of bacteria from the wound to the bloodstream (Vanderwoude et al., 2020) . *P. aeruginosa* infection of the bloodstream leads to a high mortality rate when compared to other bacterial species, due partially to the greater virulence potential of the species (Thaden et al., 2017) . Even though *P. aeruginosa* strains isolated from bacteremia cases tend to be more virulent than isolates from peripheral sites such as wounds or the respiratory tract, expression of virulence factors is not uniform between different strains of *P. aeruginosa* and is heavily influenced by the microenvironment of the infected tissue (Hickey et al., 2018) . Thus, we analyzed the virulence potential of 74 strains of *P. aeruginosa* from our culture collection, which were previously isolated from chronic wounds or bloodstream infections.

Between the vast array of tools that *P. aeruginosa* can use to harm the host, biofilm formation is one of the most important ones for chronic wound persistence (Moser et al., 2021; Tuon et al., 2022) . Estimates project that over 90% of chronic wound infections are impacted by biofilms, which contribute to healing impairment (Thi et al., 2020) . *P. aeruginosa* can form highly structured biofilms that protect the species from host defenses and environmental stresses, hindering the treatment of *P. aeruginosa* infections and contributing to colonization and persistence in human tissues (Tuon et al., 2022) . Biofilm analysis showed that, in general, adherence is lower on strains isolated from chronic wounds compared to strains isolated from the blood (p=0.0041). Using the biofilm analysis formula described by Stepanovic et al., (2000), we observed that among strains isolated from blood, 2 were weakly adherent, 6 were moderately adherent and 27 were strongly adherent. Among chronic wound strains, 2 were non-adherent, 2 were weakly adherent, 24 were moderately adherent and 11 were strongly adherent. Biofilms play a major role in persistent infection, while acute infections are usually associated with cells adopting a planktonic lifestyle (Valentini et al., 2018) . However, our evaluation of *P. aeruginosa* ability to produce biofilm showed that strains isolated from blood presented a higher biofilm-forming capacity than strains isolated from chronic wounds. A study carried out with 96 strains of different species isolated from bloodstream infections showed that most of them were weak biofilm producers. The vast majority of *P. aeruginosa* strains among those isolates, however, were strong biofilm producers (Di Domenico et al., 2021) , a result similar to what we observed in our study.

Host colonization and biofilm formation are highly influenced by motility. *P. aeruginosa* exhibits three major forms of motility: swimming, swarming, and twitching, which allow movement in aqueous, viscous, and solid surfaces, respectively. These different motility mechanisms are mediated by the species flagellum and/or type IV pilus (Yeung et al., 2012) . Analysis of different motility mechanisms showed that, overall, chronic wound strains were less motile than strains isolated from blood when it comes to swarming (p<0.0001) and twitching (p=0.0126) motility, but had no statistical difference in swimming analysis (p=0.0978). The increased ability of *P. aeruginosa* isolated from blood to move by swarming and twitching might be related to their increased biofilm formation, as both kinds of motility have been associated with biofilm formation before (O'Toole and Kolter, 1998; Shrout et al., 2006). Spearman's rank correlation analysis between biofilm and each kind of motility showed a similar association at the individual strain level. Biofilm formation was positively and significantly correlated to both swarming (r _s_ =0.412; p=0.0003) and twitching (r _s_ =0.323; p=0.0050), but not to swimming (r _s_ =0.086; p=0.4646).

Several proteolytic enzymes, such as elastase B and alkaline protease are produced by *P. aeruginosa* to colonize and persist in host tissues (Jurado-Martín et al., 2021) . In this work, we show that *P. aeruginosa* proteolytic activity on skim milk agar is significantly higher (p=0.0002) on strains isolated from blood when compared to chronic wound strains. When bacteria reach the bloodstream, it has to survive against the host innate immune system (Khan et al., 2018; Mateu-Borrás et al., 2022). The higher proteolytic activity of bloodstream infections might be an evasion mechanism that helps *P. aeruginosa* escape the host immune system. Besides contributing to biofilm formation, proteases can also contribute to the disruption of host defense mechanisms and compromise host epithelial junctions, which enable bacterial migration to tissues that are usually inaccessible (Tielen et al., 2010; Esoda and Kuehn, 2019) .

Production of pyocyanin, a green-blue pigment that plays an important role in iron metabolism (Cox 1986) , showed a high degree of variability in our analysis. Even though the mean value was lower on chronic wound strains, no significant difference (p=0.0688) in pyocyanin production was seen between the two groups. Pyocyanin is produced by 90-95% of *P. aeruginosa* strains and it has been shown to increase microbial virulence (Mavrodi et al., 2001; Gonçalves and Vasconcelos, 2021).

A longitudinal study has shown that, over time, *P. aeruginosa* virulence factors are selected against during chronic cystic fibrosis due to the genetic adaptation of the pathogen to host airways (Smith et al., 2006) . This could explain why, in our study, *P. aeruginosa* strains that colonize chronic wounds have a lower virulence potential compared to strains isolated from acute infections such as the ones in the bloodstream. However, our conclusions might still be preliminary, as further analysis (e.g., analyzing other phenotypes or strains isolated in different regions, increasing the number of strains tested, etc.) could affect the difference in virulence between these groups. For example, one caveat of our experiments was the lack of a quantitative growth rate analysis. Even though we did not see visible differences in growth between cultures, growth curves could show subtle differences in growth between strains that could have impacted some of the phenotypes tested and that should have been considered during the analysis. Also, new approaches to analyzing biofilms that don't rely on static cultures (or that use other types of surfaces that the bacteria can attach to) could show unexpected differences in the biofilm formation ability between groups. Nevertheless, we believe that our data can contribute to other studies on the virulence of *P. aeruginosa* .

## Methods

***Bacterial strains and growth conditions***

We used 74 strains of *P. aeruginosa* in this study, 35 isolated from bloodstream infections and 39 isolated from chronic wounds. The strains were part of the culture collection of the *Controle Microbiológico* laboratory of the *Universidade Federal Fluminense* , in Brazil. *P. aeruginosa* strains were routinely grown in LB culture medium at 37°C.

***Biofilm formation***

Quantification of total growth and biofilm formation was performed as described previously (do Nascimento et al., 2020) . Cells were inoculated into 96-well polystyrene plates containing LB broth and incubated at 37 °C for 24 hours. After incubation, planktonic bacteria were removed from the microplate and wells were washed with PBS (pH 7.4) three times. The plates were then dried at 60 °C for 1 hour and stained with 200 µl of 0.1% crystal violet for 30 minutes at room temperature. Excess stain was removed by rinsing the wells twice with PBS. The dye was then solubilized using 200 µl of a 95% ethanol solution, and the OD of each well was measured by spectrophotometry (SpectraMax M2e) at 570 nm.

Comparative analysis of test results was performed according to Stepanovic et al., (2000). Strains were classified into four categories based on the optical densities (OD) of biofilms. The cut-off OD value in our biofilm analysis was 0.253. Strains were considered non-adherent if OD value was lower or equal to 0.253; weakly adherent if OD value was higher than 0.253 and lower or equal to 0.505; moderately adherent if OD value was higher than 0.505 and lower or equal to 1.010; and strongly adherent if OD value was higher than 1.010.

***Motility assays***

*P. aeruginosa* motility was assessed by analysis of swimming, swarming, and twitching motility. Swimming and swarming assays were performed by touching a single colony of each *P. aeruginosa* strain with the tip of a sterile toothpick and using it to inoculate the surface of LB agar plates, followed by incubation at 37 ºC for 24 hours. The main difference between the two protocols was the agar concentration on the plates: swimming was carried out in LB with 0.3% agar and swarming in LB with 0.6% agar (Kiymaci et al., 2018) . After incubation, the plates were photographed for precise measurement of bacterial growth using Digimizer image analysis software (version 5.4.5). Swimming distance was determined by the diameter of bacterial growth and swarming was determined by the area of bacterial growth.

Twitching analysis was carried out in LB with 1% agar and was performed by touching a single colony of each *P. aeruginosa* strain with the tip of a sterile needle and using it to inoculate the bottom of the plate by stabbing the agar. After 24 hours of incubation at 37 ºC, the agar was carefully removed and 0.1% crystal violet was added to the plates to stain twitching motility (Kiymaci et al., 2018) . The plates were photographed and the diameter of the stained area was measured using Digimizer image analysis software (version 5.4.5).

***Protease activity***

Differences in proteolytic activity were assessed by touching a single colony of each *P. aeruginosa* strain with the tip of a sterile toothpick and using it to inoculate the surface of LB agar plates supplemented with 2% skimmed milk. After incubation at 37 ºC for 24 hours, proteolytic activity was evidenced by the clearance zone around the colony on equivalent-depth poured plates and was determined by the difference between colony diameter and clearance halo diameter (Kiymaci et al., 2018) using Digimizer image analysis software (version 5.4.5).

***Pyocyanin production***

Production of pyocyanin by each *P. aeruginosa* strain was evaluated as previously described, with some modifications (Ingledew and Campbell, 1969) . A single colony was inoculated on 2 mL of LB broth and incubated by shaking (200 rpm) at 37 ºC. After 48 hours, 1 ml of chloroform was added to the bacterial culture and homogenized in a vortex for 1 minute. Then, the chloroform layer was transferred to a 1.5 ml tube and centrifuged at 13,000 rpm for 2 minutes. After centrifugation, 20 µl was collected from the lower phase and the production of pyocyanin was quantified in a spectrophotometer (Ultrospec 2000 - Pharmacia Biotech) using a 50 µl quartz cuvette at an optical density of 690 nm.

***Statistical analysis***

Results for each phenotypic assay were obtained from three independent replicates and the data generated were analyzed using GraphPad Prism version 8.0.2 (GraphPad Software, San Diego, California USA). Mann-Whitney test was used to compare the two groups, with significance established by a p-value lower or equal to 0.05. Spearman's rank correlation was used to analyze the correlation between biofilm and motility phenotypes.
